# Computed tomography fluoroscopy-guided percutaneous biopsy of pulmonary nodules ≤ 10 mm: retrospective analysis of procedures performed during the COVID-19 pandemic

**DOI:** 10.1590/0100-3984.2022.0062-en

**Published:** 2023

**Authors:** Thiago Franchi Nunes, Riccardo Inchingolo, Cristina Faria Kikuti, Bernardo Bacelar de Faria, Cezar Augusto Vendas Galhardo, João Ricardo Filgueiras Tognini, Edson Marchiori, Bruno Hochhegger

**Affiliations:** 1 Hospital Universitário Maria Aparecida Pedrossian da Universidade Federal de Mato Grosso do Sul (HUMAP-UFMS), Campo Grande, MS, Brazil.; 2 Ospedale Generale Regionale Francesco Miulli, Acquaviva delle Fonti, Puglia, Italy.; 3 Laboratório Scapulatempo, Campo Grande, MS, Brazil.; 4 Núcleo Integrado de Oncologia, Hospital Unimed Campo Grande, Campo Grande, MS, Brazil.; 5 Universidade Federal de Mato Grosso do Sul (UFMS), Fundação de Ensino e Pesquisa Miguel Couto da Unimed Campo Grande, Campo Grande, MS, Brazil.; 6 Universidade Federal do Rio de Janeiro (UFRJ), Rio de Janeiro, RJ, Brazil.; 7 Pontifícia Universidade Católica do Rio Grande do Sul (PUCRS), Porto Alegre, RS, Brazil.

**Keywords:** Percutaneous biopsy, Lung, Fluoroscopy, Tomography, X-ray computed, Pulmonary nodule

## Abstract

**Objective:**

To evaluate the diagnostic performance of computed tomography (CT) fluoroscopy-guided percutaneous transthoracic needle biopsy (PTNB) in pulmonary nodules ≤ 10 mm during the coronavirus disease 2019 pandemic.

**Materials and Methods:**

Between January 1, 2020 and April 30, 2022, a total of 359 CT fluoroscopy-guided PTNBs were performed at an interventional radiology center. Lung lesions measured between 2 mm and 108 mm. Of the 359 PTNBs, 27 (7.5%) were performed with an 18G core needle on nodules ≤ 10 mm in diameter.

**Results:**

Among the 27 biopsies performed on nodules ≤ 10 mm, the lesions measured < 5 mm in four and 5-10 mm in 23. The sensitivity and overall diagnostic accuracy of PTNB were 100% and 92.3%, respectively. The mean dose of ionizing radiation during PTNB was 581.33 mGy*cm (range, 303-1,129 mGy*cm), and the mean biopsy procedure time was 6.6 min (range, 2-12 min). There were no major postprocedural complications.

**Conclusion:**

CT fluoroscopy-guided PTNB appears to provide a high diagnostic yield with low complication rates.

## INTRODUCTION

Lung cancer is the second most common cancer in Brazil and, since 1985, the most common cancer worldwide, in terms of incidence and mortality. It accounts for approximately 13% of all new cases of cancer, and only 16% of patients with lung cancer are diagnosed at an early stage, for which the five-year survival rate is 56%^(1)^.

Because of the wide availability of imaging examinations, especially computed tomography (CT), and of low-dose CT screening protocols for early lung cancer, the number of new diagnoses of pulmonary nodules is increasing and the size at which these pulmonary nodules are being detected has decreased^(2-6)^.

Although CT fluoroscopy-guided percutaneous transthoracic needle biopsy (PTNB) of pulmonary nodules has been established as a safe diagnostic procedure, with values of sensitivity and specificity > 90%^(7)^, its accuracy has been shown to decrease when the target lesion is ≤ 10 mm^(8)^.

Although fluoroscopy has been the imaging method most often utilized to guide PTNB, it has some disadvantages, including difficulty in visualizing lesions ≤ 10 mm and those located adjacent to vascular or mediastinal structures in orthogonal planes^(9)^. The aim of this study was to evaluate the diagnostic performance of CT fluoroscopy-guided PTNB in pulmonary nodules ≤ 10 mm during the coronavirus disease 2019 (COVID-19) pandemic.

## MATERIALS AND METHODS

This work was approved by the local committee for research ethics and for the administration of education and research (Reference no. 46872821.7.0000.0021). Data were collected retrospectively from the electronic medical records of patients who underwent CT fluoroscopy-guided PTNB of pulmonary nodules ≤ 10 mm between January 1, 2020 and March 30, 2022 at a tertiary interventional radiology center in the city of Campo Grande, MS, Brazil. Patients were assigned numbers to ensure that their information would remain confidential. All of those patients had been referred to the interventional radiology department for biopsy of pulmonary nodules suspected of malignancy. As a routine procedure at the tertiary care center where we perform PTNB, a multidisciplinary discussion group (tumor board) is convened. The tumor board includes professionals from at least the following specialties: pulmonology, thoracic surgery, clinical oncology, infectology, and pathology.

### Technical description of CT fluoroscopy-guided biopsy

An interventional radiologist with more than 10 years of experience in CT fluoroscopy-guided PTNB performed all of the biopsies, using a 128-slice CT scanner (Optima CT660 W; GE Healthcare, Chicago, IL, USA). Patient positioning and the choice of biopsy needle type were left to the discretion of the interventional radiologist. After the acquisition of thick (5-mm) slices, the patient was placed in the supine, prone, or lateral position. The nodule was then located and demarcated on the skin by using an electronic grid and the CT gantry lights, after which the needle entry site marked on the skin was prepared and covered in a sterile manner. All of the patients received local anesthesia and conscious sedation.

**Figure 1:**
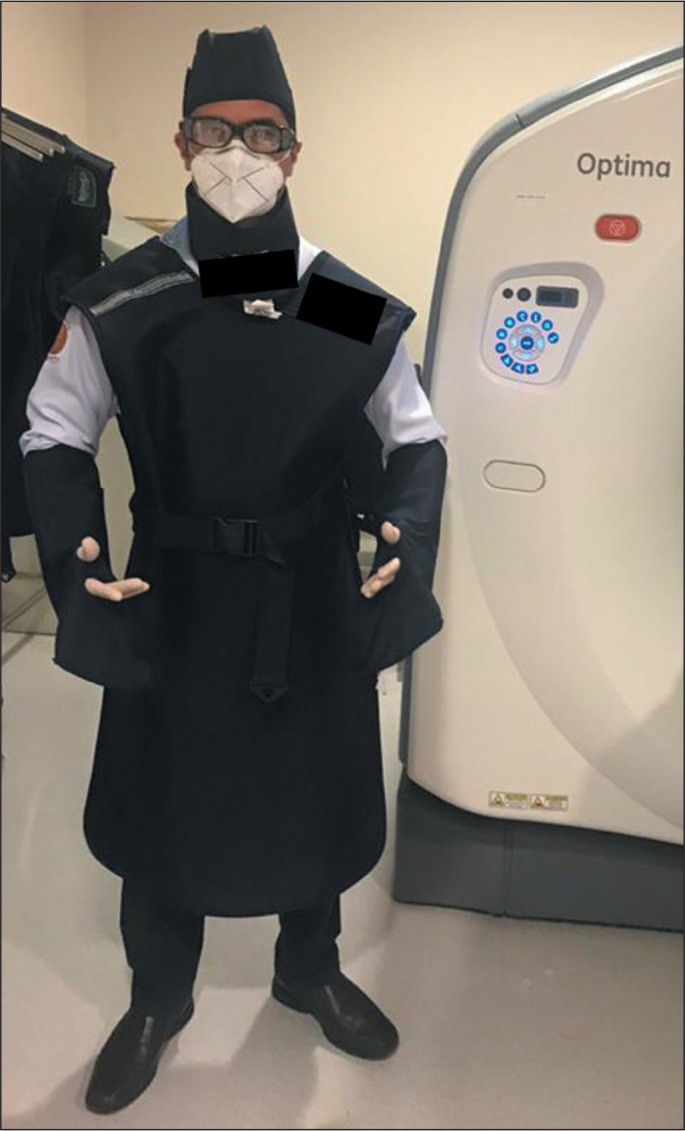
Example of personal protective equipment for radiation protection, demonstrating the preparation of the physician-operator (interventional radiologist) for CT fluoroscopy-guided biopsy procedures: lead cap, eyewear, thyroid protection, lead apron, and gloves.

All CT fluoroscopy-guided PTNBs were performed by using a self-triggering biopsy gun with an 18G × 16 cm Tru-Cut coaxial needle (Magnum; Bard Peripheral Vascular, Tempe, AZ, USA). The coaxial needle was advanced up to the edge of the lesion in accordance with the CT fluoroscopy protocol and all institutional radiation safety protocols (Figure 1). After the fragments had been removed and the coaxial needle had been advanced to a position adjacent to the target lesion, a CT scan was acquired to assess immediate complications (i.e., pneumothorax or extensive alveolar hemorrhage). After the procedure, patients were monitored in the interventional radiology recovery room. If there were no signs of complications in the immediate postprocedural period, there was no need to obtain an X-ray or CT scan of the chest before hospital discharge, which typically occurred 2-4 hours after the end of the procedure.

As per our protocol, at least two biopsy fragments were removed from each pulmonary nodule and immersed in formalin solution. On the basis of the multidisciplinary discussion, additional fragments were sent to the laboratory, either “dry” or in saline solution, for specific analyses (culture, GeneXpert rapid molecular test, etc.).

### Radiation dose and procedure time

The following settings were used when acquiring the CT fluoroscopic images: scan speed, 0.75 s/rotation (360°); tube voltage, 120 kVp; tube current, 20 mAs; and collimation, 5 mm. Radiation doses and procedure times were recorded in all cases. The patient skin doses (in mGy*cm) were measured automatically by the CT scanner. The total procedure time was defined as the time between patient entry into the CT room and withdrawal of the biopsy needle.

### Complications

Complications, including laminar pneumothorax, pneumothorax requiring chest tube insertion, hemoptysis, and other rare complications, were assessed by reviewing the records of the examinations. The criterion adopted at our institution for chest tube placement was pneumothorax (if symptomatic, occupying more than 40% of the hemithorax, or both). The chest tube was kept in place until the air leak ceased. Major complications were defined as situations in which the patient required an additional surgical procedure, such as drainage in cases of pneumothorax, in which there was prolongation of the hospital stay (e.g., due to hemoptysis with hemodynamic instability or alveolar hemorrhage involving more than one lung lobe), or in which the patient died.

### Histopathological findings

**Figure 2:**
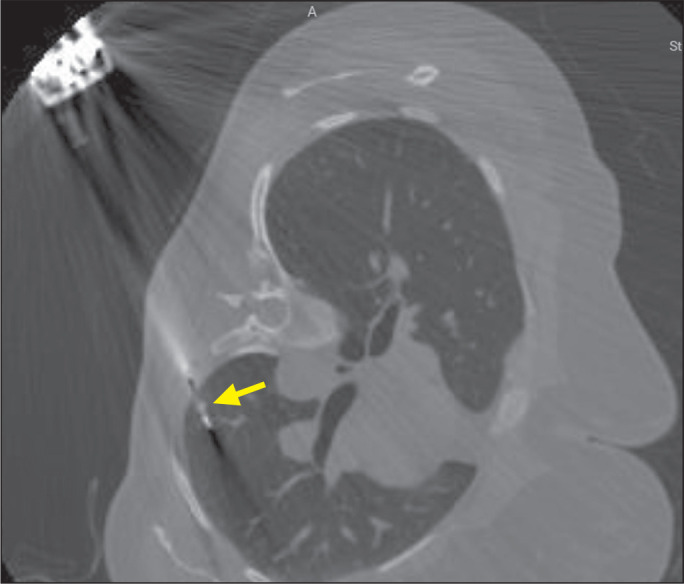
A 45-year-old woman with a history of breast cancer and emergence of a pulmonary nodule in the subpleural region of the left lower lobe, measuring 2 mm, in the left lateral position (ipsilateral to the lesion). The nodule was located at a depth of 5 mm, the needle was positioned at an angle of 45° in relation to the pleura, and two fragments of the nodule were removed. The total procedure time was 7 min, and the estimated radiation dose was 554 mGy*cm.

**Figure 3:**
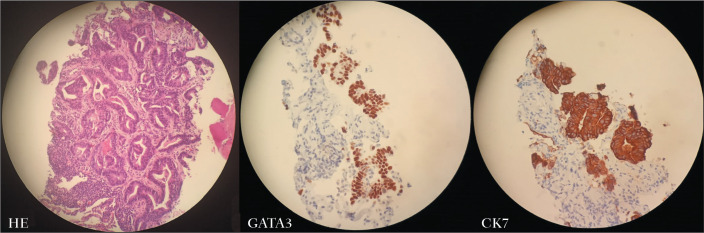
Lung biopsy fragment obtained from the patient depicted in Figure 2, showing carcinoma with a tubuloacinar pattern (hematoxylin-eosin staining; magnification, ×20). Immunohistochemistry revealed expression of GATA3 and CK7, corroborating the origin of the neoplasm in the breast.

**Figure 4:**
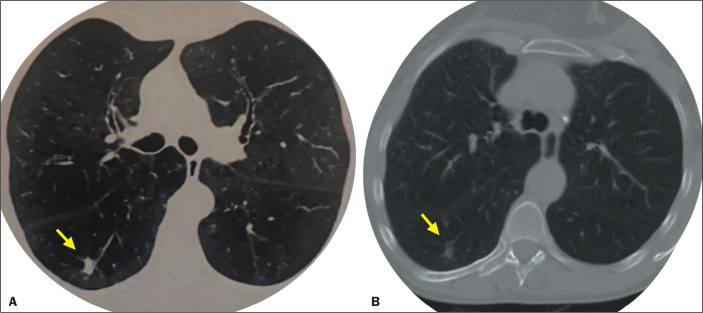
A 67-year-old male patient with a spiculated nodule in the upper segment of the right lower lung lobe. A: CT scan acquired on the day of the percutaneous biopsy, showing a lesion measuring 10 mm. B: CT scan acquired three months after the procedure, showing a significant reduction in the size of the nodule (to 5 mm), confirming the histopathological findings, and corresponding to the clinical course.

The final histological results were aggregated and classified as diagnostic or nondiagnostic. Findings of a malignant nodule or a specific benign nodule were considered diagnostic, whereas those of atypical cells, nonspecific benignity, or insufficient sample were considered nondiagnostic. Nonspecific benign diagnoses were further evaluated, and the final categorization (diagnostic vs. nondiagnostic) was based on follow-up imaging findings (Figures 2, 3, and 4) or on an additional biopsy.

### Statistical analysis

Categorical variables are expressed as absolute and relative frequencies, whereas quantitative variables are expressed as mean and standard deviation (for those with normal distribution) or as median and interquartile range (for those with asymmetric distribution). The Shapiro-Wilk test was used in order to assess the normality of the data distribution.

## RESULTS

Between January 1, 2020 and April 30, 2022, a total of 359 CT fluoroscopy-guided PTNBs were performed on lung lesions measuring between 2 mm and 108 mm. Of those 359 procedures, 27 (7.5%) were performed on nodules ≤ 10 mm.

The 27 pulmonary nodules ≤ 10 mm submitted to PTNB were in 27 patients, of whom 18 (66.7%) were women. The mean age was 60.1 ± 15.2 years. The overall results, together with data related to the biopsy technique and the characteristics of the nodules, are shown in Table 1.

**Table 1:** Demographic and clinical characteristics of the patients (N = 27), together with the characteristics of the nodules and of the intervention.

Characteristic	Values
Age (years), mean ± SD (range)	60.1 ± 15.2 (30-87)
Female, n (%)	18 (66.7)
Emphysema, n (%)	
Yes	10 (37.0)
No	17 (63.0)
Nodule characteristics	
Size (mm), mean ± SD (range)	7.9 ± 2.1 (2-10)
Lobe, n (%)	
Upper or middle	17 (63.0)
Lower	10 (37.0)
Depth, n (%)	
0-30 mm	19 (70.4)
> 30 mm	8 (29.6)
Patient position on the CT table, n (%)	
Supine or prone	12 (44.4)
Lateral	15 (55.6)
Number of fragments obtained per lesion, median (IQR; range)	2 (2-3; 1-4)
Diagnosis, n (%)	
Inconclusive	2 (7.4)
Conclusive	25 (92.6)
Benign	10 (40.0)
Malignant	15 (60.0)
Needle angle in relation to the pleura, n (%)	
≤ 50°	15 (55.6)
> 50°	12 (44.4)
Minor complications, n (%)	
Pneumothorax	2 (7.4)
Hemoptysis	3 (11.1)
Intervention time (min), mean ± SD (range)	6.7 ± 2.5 (2-12)

SD, standard deviation; IQR, interquartile range.

Among the 27 biopsies performed on nodules ≤ 10 mm, the lesions measured < 5 mm in four and 5-10 mm in 23. The median (IQR) number of biopsy gun extractions was 2 (2-3). Pneumothorax occurred during CT fluoroscopy-guided PTNB in three cases (11.1%). In those cases, the treatment was manual aspiration and a change of position in the recovery room. None of the patients with pneumothorax required pleural drainage. In addition, hemoptysis occurred in four cases (14.8%), being classified as mild and self-limiting, with no need for additional treatment, in all of those cases. We observed no life-threatening complications in our study sample.

The results were inconclusive in two (7.4%) of the CT fluoroscopy-guided PTNB procedures. In one of those cases, there was insufficient histological material. In the other case, the nodule was 3 mm in diameter and was in close proximity to the heart chamber, which made it impossible to remove all of the fragments.

**Figure 5:**
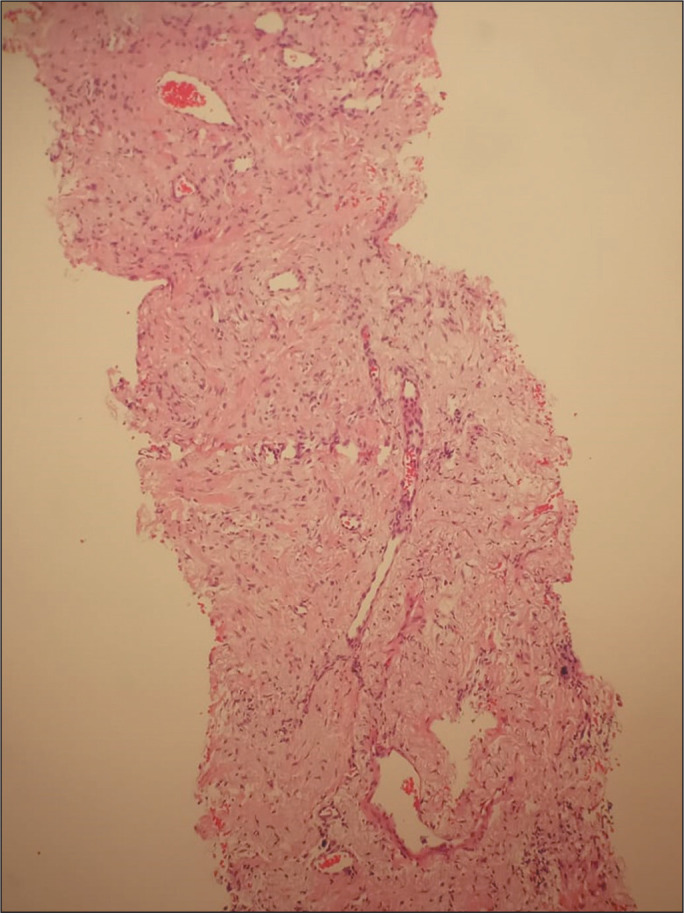
Biopsy fragment obtained from the patient depicted in Figure 4, showing fragments of lung parenchyma with septal fibrosis and discrete nonspecific chronic inflammatory infiltrate, with lymphoid aggregates, and anthracnose foci.

Among the 25 valid punctures, the histopathological diagnosis was malignant lesion in 15 (60%) and benign lesion (Figures 4 and 5) in 10 (40%) (Table 2). The results were classified as true-positive in 15 cases (55.5%), true-negative in 10 (37%), and false-negative in two (7.4%). No false-positive results were obtained. Overall, the sensitivity and diagnostic accuracy of CT fluoroscopy-guided PTNB were 100% and 92.3%, respectively.

**Table 2:** Summary of the histological diagnoses.

Diagnosis	(N = 27)
Malignancy, n (%)	15 (55.5)
Primary neoplasm	9 (33.3)
Adenocarcinoma	7 (25.9)
Squamous carcinoma	2 (7.4)
Metastasis	6 (22.2)
Benignity, n (%)	10 (37.0)
Nonspecific inflammation	3 (11.1)
Tuberculosis	1 (3.7)
Fungus	5 (18.5)
Cryptococcosis	2 (7.4)
Histoplasmosis	3 (11.1)
Chondroma	1 (3.7)
Inconclusive, n (%)	2 (7.4)

The mean dose of ionizing radiation during the CT fluoroscopy-guided PTNB procedures was 581.33 mGy*cm (range, 303-1,129 mGy*cm). The mean procedure time was 6.6 min (range, 2-12 min).

No major complications were observed after any of the procedures. However, minor complications were observed in seven patients (25.9%): laminar pneumothorax in three (11.1%) and self-limiting hemoptysis in four (14.8%).

## DISCUSSION

We evaluated the diagnostic results of 27 CT fluoroscopy-guided PTNBs of pulmonary nodules ≤ 10 mm performed at our tertiary care referral center during the COVID-19 pandemic. Although the use of percutaneous biopsy for pulmonary nodules is established as a safe diagnostic procedure, we note that few studies have reported the diagnostic performance of fluoroscopy plus CT in nodules ≤ 10 mm. To our knowledge, there have also been few studies describing the radiation dose during CT fluoroscopy-guided biopsy collection and the procedure time, factors considered quite important for interventional radiology practice and especially radiation protection.

In addition to the early detection of lung cancer with low-dose CT screening protocols, it has been observed not only that nodules are being detected earlier but also that cancer is being detected in smaller and smaller nodules^(2.3)^. Tsukada et al.^(8)^ demonstrated that pulmonary nodule size is a determining factor for the diagnostic accuracy of CT fluoroscopy-guided PTNB, that accuracy being lower for nodules < 15 mm.

In the present study, CT fluoroscopy-guided PTNB had a sensitivity and accuracy of 100% and 92.3%, respectively. In a study involving 305 biopsy procedures, Choi et al.^(10)^ found the overall sensitivity, specificity, positive predictive value, and negative predictive value of percutaneous CT-guided biopsy to be 93.1%, 98.8%, 99.3%, and 88.0%, respectively, for the diagnosis of malignancy, with a diagnostic accuracy of 95.0%. Wallace et al.^(11)^ reported that CT-guided fine-needle aspiration biopsy had an accuracy of 87.7% in a sample of 57 pulmonary nodules ≤ 10 mm. In a sample of 55 pulmonary nodules ≤ 10 mm, Ng et al.^(12)^ reported that CT-guided fine-needle aspiration biopsy had an accuracy of 78.8%. Hiraki et al.^(13)^ reported that fluoroscopy-guided Tru-Cut needle biopsy had an accuracy of 92.7% in a sample of 151 pulmonary nodules ≤ 10 mm.

In a prospective study of 875 percutaneous CT-guided lung biopsy procedures, Ruud et al.^(14)^ found that the predictors of postprocedural pneumothorax were the presence of pulmonary emphysema, target lesion size < 20 mm, longer needle time, repositioning of the coaxial needle with a new insertion through the pleura, insertion of the needle through the interlobar fissure, and shorter distance to the pleura^(14)^. We believe that the low incidence of pneumothorax in our study was due, at least in part, to the significantly shorter procedure times afforded by CT fluoroscopy (mean procedure time of 6.6 min). In our study sample, there were no cases of pneumothorax that required percutaneous drainage.

In comparison with the conventional CT-guided version, PTNB guided by CT fluoroscopy has the advantage of providing real-time visualization, which can facilitate the insertion of the needle into the lesion, thus reducing the procedure time and the number of times the needle has to be inserted. In a sample of 25 pulmonary nodules ≤ 10 mm, Yamagami et al.^(15)^ also demonstrated that CT fluoroscopy-guided biopsy provided high accuracy (88%). In the practice of conventional CT-guided biopsy, interventional radiologists require the assistance of another technician, as well as having to wait for the reconstruction of the images after digitization, during which time the patient can move and breathe, thus changing the position of the needle tip in relation to the target lesion and making the procedure more difficult. In contrast, the use of CT fluoroscopy allows interventional radiologists to obtain images and monitor the advance of the needle to its target in real time.

The mean number of nodule fragments collected in our study was 2.6 (range, 1-4), a number considered adequate for preparing slides and performing the complementary immunohistochemical studies needed in order to make an accurate diagnosis. For small lesions, pathologists recommend that the sample be separated into two vials, making it possible to create two blocks and optimize the use of the material.

The mean radiation dose employed in our sample (581.33 mGy*cm) was considered appropriate by the engineering and physics divisions of the diagnostic imaging sector. A major concern of our team was radiation protection for the team in the room (interventionist and anesthesiologist). Appropriate personal protective equipment for radiation protection, such as caps, eyewear, aprons, thyroid shields, and gloves, as well as a protective curtain positioned between the gantry and the body of the interventional radiologist, were used in the procedures performed.

We believe that the biggest problem related to the COVID-19 pandemic and interventional radiology procedures, especially those performed electively, was the limited availability of beds, not only in Brazil, but around the world. For the continuity of CT-guided percutaneous biopsy of lung lesions in a hospital environment, it is essential to change and adapt the protocols for interventional procedures. The potential risks of severe conditions in COVID-19 patients with various types of cancer were calculated in a study conducted by Dai et al.^(16)^. The authors compared different types of cancer and found lung cancer to be the most common (affecting 20.95% of the patients), followed by gastrointestinal cancer, breast cancer, thyroid cancer, and hematologic cancer. They also found that lung cancer patients had the second highest level of risk, with a mortality rate of 18.18% and an intensive care unit admission rate of 27.27%, as well as developing severe/critical symptoms in 50% of cases and requiring mechanical ventilation in 20% of cases.

Our study has some limitations. It was a single-center study, with a small number of cases, and all of the procedures were performed by the same interventional radiologist. However, we believe that we obtained a representative sample that allowed us to value the results obtained and to consider the option of CT fluoroscopy-guided procedures when planning a percutaneous biopsy of lung lesions ≤ 10 mm. The next steps for this line of research would be evaluation of the cost-effectiveness of the technique in comparison with conventional techniques and of the radiation doses received by the operating physician compared with those received during other fluoroscopy-guided techniques such as angiography.

In conclusion, CT fluoroscopy-guided PTNB performed with an 18G core needle appears to have a high diagnostic yield. The procedure also seems to have low complication rates.
